# Purinergic and Energy Metabolism Disruption in Oxidative Stress-Mediated Immunotoxicity Induced by Aflatoxin B_1_ and Fumonisin B_1_

**DOI:** 10.3390/jof12070520

**Published:** 2026-07-15

**Authors:** Verónica S. Mary, Pilar A. Velez, Sol Quiroz, Sofía A. Díaz Iriso, Candelaria Amilibia, Héctor R. Rubinstein, Martín G. Theumer

**Affiliations:** 1 Departamento de Bioquímica Clínica, Facultad de Ciencias Químicas, Universidad Nacional de Córdoba (UNC), Córdoba X5000HUA, Argentina; 2 Centro de Investigaciones en Bioquímica Clínica e Inmunología (CIBICI, UNC-CONICET), Córdoba X5000HUA, Argentina

**Keywords:** adenosine, ATP, aflatoxin B_1_, energy status, fumonisin B_1_, immunotoxicity, purinergic metabolism, natural killer cells, oxidative stress, Th1 responses

## Abstract

Aflatoxin B_1_ (AFB_1_) and fumonisin B_1_ (FB_1_) are cereal-contaminating mycotoxins; co-exposure to these compounds is associated with hepatocellular carcinoma, although the underlying immunological mechanisms remain unclear. This study aimed to investigate the impact of AFB_1_ and FB_1_, individually and combined, on immune cell function, focusing on Th1-associated cytokines, cytotoxic capacity, oxidative stress involvement, and energy and purinergic metabolism. Rat splenocytes and intrahepatic leukocytes were exposed to AFB_1_ (20 μM), FB_1_ (10 μM), or a mixture of both (MIX), in the presence or absence of catalase. Cytokines and phenotypes were assessed by flow cytometry and ELISA, cytotoxicity by co-culture assays, and intra/extracellular adenine nucleotides and purines by HPLC. The MIX significantly impaired Th1-like differentiation and reduced IFNγ and TNFα production, FAS-L expression and cytolytic activity while increasing IL4 production, indicating a shift toward a Th2-like profile. These effects were prevented by catalase, supporting a key role for oxidative stress. Co-exposure disrupted intracellular energy balance, decreasing adenosine triphosphate (ATP) levels, ATP/ADP ratios, and total adenine nucleotide pools. It also enhanced purine degradation, extracellular ATP release and adenosine accumulation, suggesting altered purinergic signaling. Consequently, AFB_1_-FB_1_ co-exposure disrupts immune function through interconnected redox and metabolic mechanisms, promoting an immunosuppressive environment that may favor tumor progression.

## 1. Introduction

Mycotoxins are common natural contaminants of cereals and pose a major risk to human and animal health due to their high worldwide incidence [1,2]. Among them, aflatoxin B_1_ (AFB_1_) and fumonisin B_1_ (FB_1_), mainly produced by *Aspergillus* and *Fusarium* species, respectively, are considered particularly relevant because of their high prevalence, toxicity, and frequent co-occurrence in grains. This co-occurrence has been associated with an increased incidence of hepatocellular carcinoma (HCC) [3,4,5,6,7].

The development of HCC in humans has been primarily attributed to genotoxic mechanisms, in which AFB_1_ acts as a mutagenic agent [8], whereas FB_1_ has been proposed to function as a tumor promoter [9]. However, immune system dysregulation induced by the individual or combined action of these mycotoxins has been proposed as an additional mechanism contributing to disease progression [10,11], consistent with the established role of immune dysfunction in HCC [12]. While it is well established that AFB_1_ and FB_1_ can induce immunomodulatory and immunotoxic effects [4,6,13], the immunological consequences and underlying mechanisms of combined AFB_1_-FB_1_ exposure remain insufficiently characterized.

In this context, Th1 cells and their associated cytokines, particularly IFNγ, TNFα, and IL2, play a central role in antitumor immunity in HCC by promoting the activation of effector cells such as M1 macrophages and cytotoxic populations, including natural killer (NK) cells, cytotoxic CD8^+^ T lymphocytes, and natural killer T (NKT) cells. Disruption of Th1 responses favors the establishment of an immunosuppressive microenvironment and tumor immune evasion [12,14]. Consistently, HCC patients exhibit reduced levels of Th1 cytokines and increased Th2-associated cytokines including (IL4, IL5, and IL10), reflecting a shift toward a Th2-biased immune profile that is associated with poor prognosis [12,15,16]. In addition, immune evasion in the HCC microenvironment is reinforced by the accumulation of metabolic intermediates such as adenosine (ADO), an adenosine triphosphate (ATP)-derived metabolite that impairs Th1 cell function and promotes regulatory T-cell (Treg) expansion, thereby fostering an immunosuppressive milieu [12].

Oxidative stress is a critical factor in HCC pathogenesis promoting genomic instability and modulating the tumor immune microenvironment. Sustained redox imbalance may interfere with key signaling pathways that regulate immune cell function, contributing to the impairment of Th1-mediated responses and favoring Treg expansion [17]. In addition, these effects may converge with metabolic immunoregulatory mechanisms, including ATP production and ADO signaling, suggesting a functional interplay between redox status, cellular energy metabolism, and purinergic pathways in the establishment of an immunosuppressive milieu [18,19,20,21,22].

Increasing evidence has shown that AFB_1_ and FB_1_, individually and particularly in mixtures, generate reactive oxygen species (ROS) leading to oxidative stress [13,23,24,25] and likely alterations in cellular signaling pathways involved in immune regulation [10,13]. However, the contribution of oxidative stress to the functional impairment of specific immune responses, such as Th1 polarization and cytotoxic activity, remains incompletely understood in the context of combined AFB_1_-FB_1_ exposure.

In addition to redox imbalance, emerging evidence indicates that immune cell function is tightly linked to cellular energy metabolism. The activation, proliferation and effector functions of immune cells depend on tightly regulated ATP production and adenine nucleotide homeostasis [22], as they use ATP as their primary energy source for metabolic reactions. The ratios of ATP, adenosine diphosphate (ADP), and adenosine monophosphate (AMP) roughly reflect cellular energy status [26,27]. Metabolic and/or oxidative stress induces significant changes in both the levels and relative proportions of these nucleotides, as well as in their turnover and catabolism to inosine, hypoxanthine, and xanthine [20,26]. Furthermore, alterations in intracellular energy metabolism may have direct consequences on extracellular purinergic signaling. Under conditions of cellular stress, ATP can be released into the extracellular space and subsequently degraded to adenosine (ADO), a potent immunomodulatory molecule [28].

In this regard, some studies have shown that AFB_1_ may alter intracellular ATP levels or affect enzymes involved in purinergic metabolism, whereas evidence for FB_1_ is scarcer. Moreover, investigations in immune cells remain very limited [29,30,31], and the effects of combined exposure to these mycotoxins, as well as their potential interactions, on intracellular energy status and purinergic signaling remain largely unexplored.

To the best of our knowledge, this is the first study to simultaneously investigate the contribution of oxidative stress, intracellular energy metabolism, and extracellular purinergic signaling to the immunotoxic effects induced by combined AFB_1_-FB_1_ exposure. In addition, the study examines whether interactions between these mycotoxins may result in additive or synergism-consistent responses across different immune and metabolic endpoints. By integrating these mechanisms, the present work provides a novel framework for understanding how mycotoxin co-exposure may promote immune dysfunction and contribute to tumor-promoting conditions beyond the well-established genotoxic effects of these contaminants.

Therefore, the aim of this study was to investigate, in vitro, the effects of AFB_1_ and FB_1_, individually and in combination, on the function of rat splenic mononuclear cells and intrahepatic leukocytes, with particular attention to Th1-type responses and cytotoxic capacity, and to determine the contribution of oxidative stress to their potential immunotoxic effects. In parallel, the study aimed to evaluate the energy status of immune cells and their extracellular purinergic profile, and to characterize the toxicological interactions between these mycotoxins across the assessed parameters.

## 2. Materials and Methods

### 2.1. Chemicals

RPMI-1640 and DMEM media, and heat-inactivated fetal bovine serum (FBS) were purchased from Gibco Laboratories (Buenos Aires, Argentina), and Bradford reagent was obtained from Bio-Rad Laboratories (Buenos Aires, Argentina). 3-(4,5-dimethylthiazol-2-yl)-2,5-diphenyltetrazolium bromide (MTT), ADO, ADP, AFB_1_, ammonium chloride, AMP, ATP, brefeldin A, bovine serum albumin (BSA), catalase (CAT), dimethyl sulfoxide (DMSO), FB_1_, gentamicin, hypoxanthine (HPX), inosine (INO), ionomycin, L-glutamine, lysis buffer, paraformaldehyde (PFA), Percoll, phorbol 12-myristate 13-acetate (PMA), trypan blue, and trypsin were purchased from Sigma-Aldrich (Buenos Aires, Argentina). The monoclonal antibodies anti-CD32, APC anti-CD3, FITC anti-NK1.1, PE anti-IFNγ, PE anti-IL4, and biotin anti-FAS-L were provided by BD Biosciences (Buenos Aires, Argentina), whereas APC anti-CD4, AF594 anti-IFNγ, and PE anti-TNFα were obtained from Invitrogen (Thermo Fisher Scientific, Buenos Aires, Argentina). PE-streptavidin and E-Fluor 670 were obtained from e-Bioscience (Thermo Fisher Scientific, Buenos Aires, Argentina). The Phosphatase Inhibitor Cocktail I and Protease Inhibitor Cocktail were purchased from TargetMol Chemicals Inc. (Boston, MA, USA). All other chemicals were provided by Sintorgan (Buenos Aires, Argentina) at the highest analytical grade available.

### 2.2. Cell Culture

Splenic mononuclear cells (SMCs), intrahepatic leukocytes (IHLs), and peritoneal cells were obtained from male Wistar inbred rats (6–8 weeks old) kept in environmentally controlled rooms with a 12 h light–dark cycle. The spleens and livers were removed aseptically from the six animals and pooled.

SMC suspensions were prepared as previously described by Mary et al. [25], seeded at a density of 10^6^ cells/mL, and cultured in complete RPMI 1640 medium (supplemented with 10% FBS, 2 mM glutamine, and 50 μg/mL gentamicin).

IHL suspensions were prepared as previously described by Shi et al. [32], with minor modifications. Briefly, livers were perfused with 20 mL of PBS containing 3% FBS, mechanically dissociated through a 200-gauge stainless steel mesh, and resuspended in PBS-3% FBS. Erythrocytes were then removed using lysis buffer, and IHLs were obtained after 20 min of centrifugation (600× *g*) on a 35% and 65% bilayer Percoll gradient. The cells were washed in PBS and cultured at a density of 10^6^ cells/mL in complete RPMI 1640 medium. Furthermore, as IHLs are enriched in NK and NKT cell populations compared with secondary lymphoid organs [33,34,35], they were stimulated with IL-12 (5 ng/mL) and IL-18 (20 ng/mL) to induce their functional activation toward a Th1-like profile.

Peritoneal cells were obtained by washing the peritoneal cavity of normal rats with cold PBS containing 0.1% FBS and 5 mM EDTA. The cells were washed, pooled, resuspended, and cultured in complete RPMI 1640 medium at a concentration of 8 × 10^6^ cells/mL. The cell suspension was enriched in adherent cells (ACs) by allowing the cells (1 mL, 8 × 10^6^ cells/mL) to adhere in 24-well plates at 37 °C and 5% CO_2_ for 3 h, after which non-adherent cells were removed by washing with warm RPMI 1640. The AC fraction obtained by adherence was predominantly enriched in macrophages, consistent with previous studies using rodent peritoneal adherent cells [36,37].

SMCs were also obtained from male inbred mice (6–8 weeks old) kept in environmentally controlled rooms with a 12 h light–dark cycle. The *Foxp3*^GFP^ knock-in mouse strains used were C57BL/6, as wild type (WT), and B6.D2N-Ahrd/J, which has a C57BL/6 background and is homozygous for the Ahr^d^/Ahr^d^ allele, expressing an aryl hydrocarbon receptor (AhR) with low-affinity (10- to 100-fold lower) for its ligands [10]. Both mouse strains were purchased from Jackson Laboratories (Bar Harbor, ME, USA). For each experiment, the spleens were removed aseptically from the six mice and pooled, and splenocyte suspensions were prepared as described above for rat SMCs.

The BRL-3A immortal rat liver cell line was obtained from the American Type Culture Collection (ATCC) (Manassas, VA, USA). This cell line was cultured in DMEM medium supplemented with 10% FBS, 2 mM glutamine, and 50 μg/mL gentamicin, and split weekly with 0.5% trypsin/0.02% EDTANa_2_. The BRL-3A cells were seeded in 24-well plates at a density of 6 × 10^4^ cells/well [24].

For all studies, immune cells were cultured in the presence or absence of 20 μM AFB_1_ dissolved in DMSO (0.07%), 10 μM FB_1_ dissolved in PBS or the two-toxin mixture (20 μM AFB_1_ + 10 μM FB_1_, MIX), at 37 °C in a humidified atmosphere with 5% CO_2_. Treatments lasted up to 48 h, depending on the experiment. The choice of doses was based on the maximum permissible levels of these mycotoxins in international food standards, the intestinal absorption of AFB_1_ and FB_1_ (about 100% and 4%, respectively) after oral administration to rats, published literature, and our previous studies [25,38,39,40]. In particular, these concentrations were previously shown to induce oxidative stress in rat splenocytes without causing marked cytotoxicity [25]. Therefore, they were selected to investigate the contribution of oxidative stress to the immunotoxic effects induced by AFB_1_ and FB_1_. In addition, control cells were treated with DMSO (0.07%, *v*/*v*).

Cell viability was studied up to 48 h of incubation with the mycotoxins or the AFB_1_ vehicle (DMSO), using the trypan blue exclusion test and the MTT assay. Cell viability at 48 h remained between 80% and 90%. These viability data suggest that the immunological changes observed under the experimental conditions employed are unlikely to be explained solely by overt cytotoxic effects.

In addition, to evaluate the contribution of oxidative stress to the effects of the individual and combined mycotoxins on immune cell functionality, the cells were pre-incubated for 2 h with catalase (1000 U/mL), an H_2_O_2_ scavenger. This enzyme was chosen because previous work demonstrated that catalase can significantly reduce AFB_1_- and FB_1_-induced biomolecular oxidative damage in rat SMCs [25].

### 2.3. Immunofluorescent Staining for Detection of Surface Antigens and Intracellular Cytokines by Flow Cytometry

One million rat immune cells (SMCs or IHLs) were incubated with anti-CD32 mAb (0.4 μL/2 × 10^5^ cells) in PBS for 30 min at 4 °C, and then stained with APC anti-CD3 mAb, FITC anti-NK1.1 mAb, biotin-conjugated anti-FAS-L mAb, and/or PE streptavidin. The corresponding isotype controls were from BD Biosciences. All staining steps were performed at 4 °C in PBS-3% FBS for 30 min in the dark. After incubation, cells were washed twice with PBS-3% FBS and then fixed overnight at 4 °C with 1% PFA.

Intracellular cytokines were detected after stimulating 10^6^ cells with 50 ng PMA and 500 ng ionomycin, in the presence of 5 ng brefeldin A, during the last 5 h of the 24 h culture period. Surface-stained and fixed cells, were permeabilized with 0.5% saponin in PBS for 15 min and blocked with 5% heat-inactivated rat serum in the saponin solution for 15 min. Cells in this buffer were incubated with PE anti-IFNγ mAb, PE anti-IL4 mAb or appropriate controls (corresponding to IgG-negative isotypes) in the dark for 30 min at room temperature. Then, cells were successively washed with the saponin solution and PBS-3% FBS, and resuspended in flow wash buffer. Finally, the stained cells were collected using a FACSCanto II flow cytometer (BD Biosciences, San Jose, CA, USA) and analyzed using WinMDI software version 2.9. The leukocyte population was gated based on forward and side light-scatter parameters. T lymphocytes were identified as CD3^+^ cells in the leukocyte gate from SMCs. NK cells were identified as NK1.1^+^ cells, and NKT cells were labeled as CD3^+^ and NK1.1^+^ cells in the leukocyte gate from IHLs. FasL expression was analyzed in T lymphocytes and is depicted as the percentage of FasL^+^ cells.

Furthermore, mouse spleen T cells were stimulated for 48 h with plate-bound antibodies against CD3 (145-2C11, 2 mg/mL) and CD28 (37.51, PV-1; 1 mg/mL), in the presence of mouse recombinant IL-12 (10 nM), to induce T cell differentiation into Th1 [41]. After the AFB_1_ and/or FB_1_ treatments described above, SMCs were stained with APC anti-CD4 mAb and a fluorescent probe for the visualization of cell viability, at 4 °C in buffer (PBS-2% FBS) for 20 min in the dark. Subsequently, cells were washed twice with this buffer and fixed at 4 °C for 20 min in the dark with BD Cytofix™. Intracellular cytokines were detected after stimulating 10^6^ cells with 50 ng PMA and 500 ng ionomycin, in the presence of BD GolgiPlug™ or BD GolgiStop™, during the 4 h before ending the culture. Surface-stained and fixed cells, were permeabilized with BD Cytoperm™ at 4 °C for 15 min in the dark, and incubated with AF594 anti-IFNγ mAb and PE anti-TNFα mAb in permeabilization buffer for 30 min at room temperature in the dark. Then, cells were washed and resuspended in PBS-2% FBS. Finally, samples were collected using FACS LSR Fortessa or FACSCanto II (BD Biosciences, San Jose, CA, USA) and data were analyzed using Flow Jo software (Version 10, BD Biosciences, Ashland, OR, USA).

### 2.4. The Tumor Necrosis Factor-α (TNFα) Secretion

The TNFα production by SMCs, IHLs, and ACs was measured in culture supernatants collected after 48 h of incubation. Concanavalin A (Con A, 5 μg/mL), IL-12 (10 ng/mL), and PMA (50 ng/mL) were used as stimuli for SMCs, IHLs, and ACs, respectively. Con A and IL-12 were added at the beginning of the cultures, whereas PMA was added 6 h before the end of the AC cultures. Cells were cultured in the presence or absence of the mycotoxin treatments and/or CAT (1000 U/mL). Supernatants were collected and stored at −80 °C until analysis. TNFα levels were measured using sandwich ELISA CytoSets according to the manufacturer’s protocol (Biosource, Camarillo, CA, USA). A capture anti-TNFα mAb in combination with biotinylated mAb was used. After washing, the plates were reacted with horseradish peroxidase streptavidin (Biosource), followed by the addition of tetramethylbenzidine (Biosource) for 5–20 min, and the reaction was stopped with sulfuric acid. Measurements were obtained using a Microplate Reader (Bio-Rad, Hercules, CA, USA) at 450 nm, and results are expressed in pg/mL. Recombinant rat TNFα was used as the standard.

### 2.5. Cytolytic Activity of Rat SMCs

To assess the cytolytic activity of spleen cells [42], the BRL-3A cells were used as targets. Briefly, the SMC suspension was divided into two groups. One group was cultured in 24-well plates at a density of 1 × 10^6^ cells/well, in RPMI 1640 medium, supplemented in the presence or absence of mycotoxins (individually or in combination), and/or CAT (1000 U/mL) for 24 h. The remaining group of SMCs (control population) and BRL-3A cells were washed twice and resuspended in sterile PBS (10 × 10^6^ cells/mL). An equal volume of sterile PBS containing eFluor-670 dye was then added to the SMC and BRL-3A suspensions to achieve final concentrations of 1 µM and 10 µM, respectively. After incubation for 10 min at 37 °C, the cells were washed three times with sterile PBS, fixed with 1% sterile PFA for 30 min at room temperature, maintained overnight at 4 °C, washed again, and resuspended in complete RPMI 1640 medium. After 24 h of incubation of the undyed SMCs with the indicated treatments, stained SMCs and BRL-3A cells were mixed at a 1:1 ratio, and 5 × 10^5^ cells/well of the mixture were added to the cultures, which were then incubated for an additional 24 h (up to 48 h total). A pool of stained SMCs and BRL-3A cells that were not cocultured with undyed SMCs served as a control. Finally, cells were acquired by flow cytometry and analyzed by comparing the ratio of target BRL-3A cells (eFluor 670^high^) to SMCs (eFluor 670^low^). Results were expressed as the percentages of cells with high fluorescence (BRL-3A target cells) relative to the control condition. Values higher than the control (1.0) indicate lower cytolytic activity, reflecting a higher percentage of surviving target cells, whereas values below 1.0 indicate a reduction in the proportion of target cells and, consequently, increased cytolytic capacity.

### 2.6. Determination of Intra- and Extracellular Adenine Nucleotides and Purine Metabolites in SMCs

Adenine nucleotides and purine metabolites were measured in the cytosolic fraction of rat SMCs exposed to the mycotoxin treatments and in culture supernatants collected after 16 h. Cells were washed with PBS and lysed with 50 mM HEPES buffer (pH 7.4) containing protease and phosphatase inhibitors. Following collection, both supernatants and cell lysates were stored at −80 °C until analysis. Protein content in the lysates was determined by the Bradford method. Samples were deproteinized with cold methanol (8:2, *v*/*v*) and centrifuged at 10,000 rpm for 2 min at 4 °C. Intracellular and extracellular levels of ATP, ADP, AMP, ADO, INO, and HPX were determined using a Hewlett Packard Series 1200 HPLC system equipped with a 20 μL injection loop and coupled to a UV–visible detector (Agilent Technologies, Santa Clara, CA, USA) set at 257 nm. Chromatographic separation was performed using a Luna 100 RP-18 column (250 × 4.6 mm, 5 μm) with a guard column of the same material (Phenomenex, Torrance, CA, USA). The mobile phase consisted of methanol and 0.05 M KH_2_PO_4_ buffer (pH 6.2) in a 1:9 ratio, delivered at a flow rate of 0.8 mL/min. Quantification of metabolites was performed by comparing the peak areas from the samples with those obtained from analytical standards of ATP, ADP, AMP, ADO, INO, and HPX (purity > 95%), using HP ChemStation Rev. A.07.01 software. Subsequently, the nucleotide ratios of interest were determined, and the adenylate energy charge (EC) was calculated according to the following equation: EC = (ATP + 0.5ADP)/(ATP + ADP + AMP) [27]. In addition, purine degradation was estimated by calculating a purine degradation index (PDI), defined as: PDI = (ADO + INO + HPX)/(ATP + ADP + AMP + ADO + INO + HPX), based on the accumulation of purine catabolites generated during ATP degradation [43,44]. This analytical approach enabled the direct and simultaneous quantification of intracellular and extracellular adenine nucleotides and purine metabolites, allowing the assessment of the actual metabolic outcome of ATP turnover. The simultaneous evaluation of both intracellular and extracellular metabolites provided a more comprehensive and mechanistic characterization of the energetic and purinergic status of immune cells.

### 2.7. Statistical Analysis

Data from these studies were obtained from a minimum of three independent experiments (*n* = 6 for each treatment) and assessed by a one-way ANOVA followed by Bonferroni’s post hoc test using GraphPad InStat software version 3.01 (La Jolla, CA, USA). Results were expressed as the mean ± standard error of the mean (SEM), with differences being considered significant at the *p* < 0.05 level.

## 3. Results

### 3.1. Effects of AFB_1_ and FB_1_ on Cytokine Production

#### 3.1.1. Modulation of IFNγ and TNFα Production by Mycotoxins and Catalase in Rat SMCs

**Figure 1:**
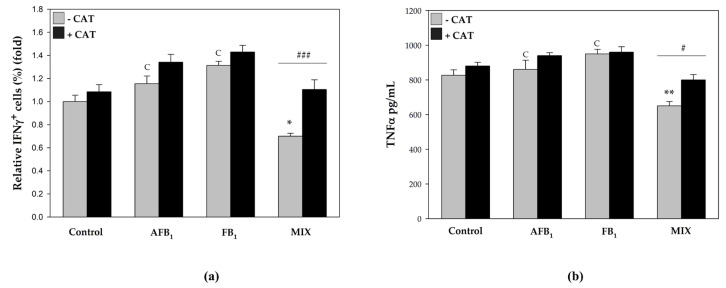
Effects of AFB_1_ and FB_1_ on IFNγ and TNFα production by rat SMCs: (**a**) Percentage of IFNγ-producing T cells, relative to the control; (**b**) Level of TNFα in SMC culture supernatants. Gray bars represent cells preincubated with catalase (+ CAT), whereas black bars represent cells without catalase treatment (− CAT). Values are expressed as mean ± SEM. * Indicates differences vs. the corresponding control. Letters indicate differences between MIX and individual mycotoxin treatments. ^#^ Indicates differences between treatments performed in the presence or absence of CAT. *^,#^ *p* < 0.05; ** *p* < 0.01; ^C,###^ *p* < 0.001.

To assess the effects of the mycotoxins on the production of cytokines relevant to antitumor immunity (such as IFNγ and TNFα) by SMCs, and to determine whether alterations in the oxidative status of these cells contribute to these effects, SMCs were preincubated with CAT and cultured in the presence or absence of AFB_1_, FB_1_, or the MIX for up to 48 h. The percentages of IFNγ-producing T cells (CD3^+^), determined by flow cytometry, and the TNFα levels obtained by ELISA in the culture supernatants are shown in Figure 1a and Figure 1b, respectively.

The results indicated that individual exposure to AFB_1_ or FB_1_ did not induce significant changes in the percentages of IFNγ-producing T cells or in TNFα secretion by SMCs. In contrast, exposure to the MIX significantly reduced both parameters compared with the control and with the effects observed for the individual mycotoxins. Moreover, in SMC cultures preincubated with CAT, the proportions of IFNγ^+^ T cells and TNFα levels were comparable to those of the control and significantly higher than those observed in MIX-treated cultures, indicating that CAT prevented the immunotoxic effects induced by the MIX and suggesting the involvement of oxidative stress in the mechanism of action of this treatment.

#### 3.1.2. Modulation of IFNγ and TNFα Production by Mycotoxins in SMCs from Mice with AhRs of Different Affinities

Previous studies demonstrated that CYP4501A activity is a source of ROS induced by AFB_1_ and/or FB_1_, and that the AhR receptor, a potent inducer of CYP4501A, is upregulated by the combination of both toxins in rat and mouse spleen and liver cells [10,24,25,45]. In addition, AhR signaling has been associated with the regulation of Th1 cytokine production [41]. Therefore, the role of AhR in the inhibitory effects of AFB_1_ and FB_1_ on Th1 cytokine production was investigated using mice expressing AhR isoforms with different ligand-binding affinities, since AhR-mediated immune responses are highly ligand-dependent. This experimental strategy has been widely used to evaluate ligand-specific AhR effects under physiological signaling conditions [46].

**Figure 2:**
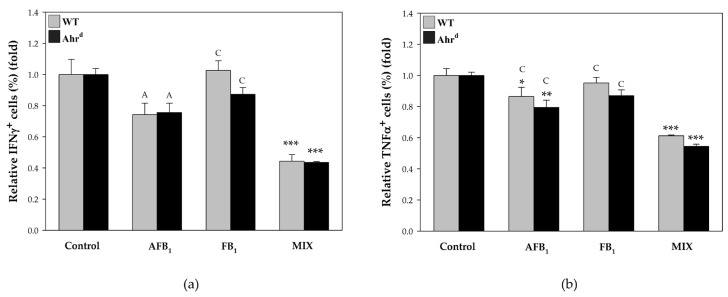
Effects of AFB_1_ and FB_1_ on Th1-like cell differentiation from mouse SMCs: (**a**) Percentage of IFNγ-producing CD4^+^ T cells; (**b**) Percentage of TNFα-producing CD4^+^ T cells. Data are expressed relative to the control. Gray bars represent cells from wild-type (WT) mice, whereas black bars represent cells from Ahr^d^ mice expressing a low-affinity AhR ligand-binding phenotype. Values are expressed as mean ± SEM. * Indicates differences vs. the corresponding control. Letters indicate differences between the MIX and individual mycotoxin treatments. *^,A^ *p* < 0.05; ** *p* < 0.01; ***^,C^ *p* < 0.001.

The percentages of Th1-like cells (CD4^+^ IFNγ^+^ and CD4^+^ TNFα^+^), determined by flow cytometry, were normalized to the values obtained in cultures treated with IL12 alone (a Th1 cell differentiation–stimulating cytokine, 100%) and are shown in Figure 2. Treatment with individual AFB_1_ and, especially, the MIX, significantly reduced the percentages of Th1-like cells producing IFNγ and/or TNFα compared with the control, in cultures derived from WT mice, strongly suggesting that these treatments impair murine Th1 cell differentiation. Moreover, similar immunotoxic effects were observed in SMC cultures derived from mice expressing a low-affinity AhR for its ligands, indicating that AhR signaling is not involved in these effects induced by AFB_1_ alone or in combination with FB_1_.

#### 3.1.3. Modulation of TNFα Secretion by Mycotoxins in Rat ACs

Consistent with the findings in murine SMC cultures, incubation of ACs with AFB_1_, either alone or in combination with FB_1_, resulted in significantly lower TNFα levels compared to control cultures. In addition, TNFα titers in the supernatants of ACs cultured in the presence of the individual mycotoxins were significantly different from those observed in MIX-treated cultures (Figure S1).

#### 3.1.4. Modulation of TNFα, IFNγ and IL4 Production by Mycotoxins and Catalase in Rat IHLs

To evaluate the effects of mycotoxins on cytokine production and to assess the contribution of the oxidative action of AFB_1_ and FB_1_, IHLs stimulated with IL12/IL18 to induce functional activation of NK and NKT cells toward a Th1-like profile were preincubated or not with CAT and subsequently exposed to AFB_1_, FB_1_, or their combination (MIX) for up to 48 h.

**Figure 3:**
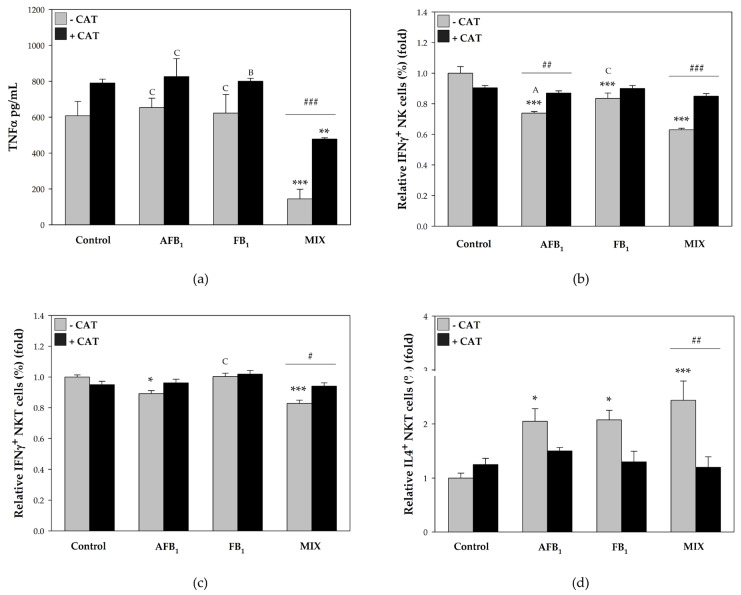
Effects of AFB_1_ and FB_1_ on TNFα, IFNγ, and IL4 production by rat IHLs: (**a**) Levels of TNFα in IHL culture supernatants; (**b**) Percentage of IFNγ-producing NK cells; (**c**) Percentage of IFNγ-producing NKT cells; (**d**) Percentage of IL4-producing NKT cells. All percentage data are expressed relative to the control. Gray bars represent cells preincubated with catalase (+ CAT), whereas black bars represent cells without catalase treatment (− CAT). Values are expressed as mean ± SEM. * Indicates differences vs. the corresponding control. Letters indicate differences between the MIX and individual mycotoxin. ^#^ Indicates differences between treatments performed in the presence or absence of CAT. *^,A,#^ *p* < 0.05; **^,B,##^ *p* < 0.01; ***^,C,###^ *p* < 0.001.

Similar to the observations in rat SMCs, no changes in TNFα levels were detected by ELISA in IHL cultures exposed to AFB_1_ or FB_1_ alone compared to the control (Figure 3a). However, cultures treated with the MIX showed a significant reduction in TNFα release relative to the control and to the individual mycotoxins. In addition, when IHLs were cultured in the presence of CAT and the MIX, a significant restoration of secreted TNFα levels was observed (Figure 3a). Flow cytometry analysis showed that individual and combined mycotoxin treatments significantly reduced the percentage of IFNγ-producing NK (NK1.1^+^) cells, with the MIX causing the greatest decrease (Figure 3b). In contrast, preincubation with CAT preserved IFNγ^+^ NK cell percentages at levels comparable to those of the control, which were significantly higher than those observed in cultures treated with MIX or AFB_1_ alone (Figure 3b). Moreover, AFB_1_ alone and the MIX also impaired the activation of NKT cells toward a Th1-like profile, as evidenced by a significant reduction in the percentages of IFNγ-producing NKT (CD3^+^ NK1.1^+^) cells, whereas FB_1_ did not alter the proportion of these cells (Figure 3c). Consistent with the effects observed in NK cells, CAT prevented the decrease in IFNγ^+^ NKT cell percentages induced by AFB_1_ and MIX (Figure 3c), restoring these percentages to levels comparable to those observed in the control group. In addition, the proportion of IFNγ^+^ NKT cells was significantly higher in IHL cultures preincubated with CAT and exposed to the MIX, than in those treated with the MIX alone. Taken together, these findings indicate that CAT counteracts the immunotoxic effects induced by individual and combined mycotoxin exposure on the functional activation of the IHLs toward a Th1-like profile, supporting the involvement of oxidative stress in the underlying mechanisms of these responses.

On the other hand, all mycotoxin treatments significantly increased the percentage of IL4–producing NKT cells. In contrast, preincubation with CAT prevented the effects induced by these treatments, as combined exposure to CAT and mycotoxins (either individually or in combination) did not significantly alter the proportion of IL-4^+^ NKT cells compared with the control group (Figure 3d). Moreover, preincubation with CAT significantly reduced the percentage of IL-4^+^ NKT cells induced by the MIX. Taken together, these results suggest that oxidative stress triggered by AFB_1_ alone or in combination with FB_1_ promotes the polarization of these cells toward a Th2-like profile.

### 3.2. Effects of AFB_1_ and FB_1_ on Cytotoxic Capacity

**Figure 4:**
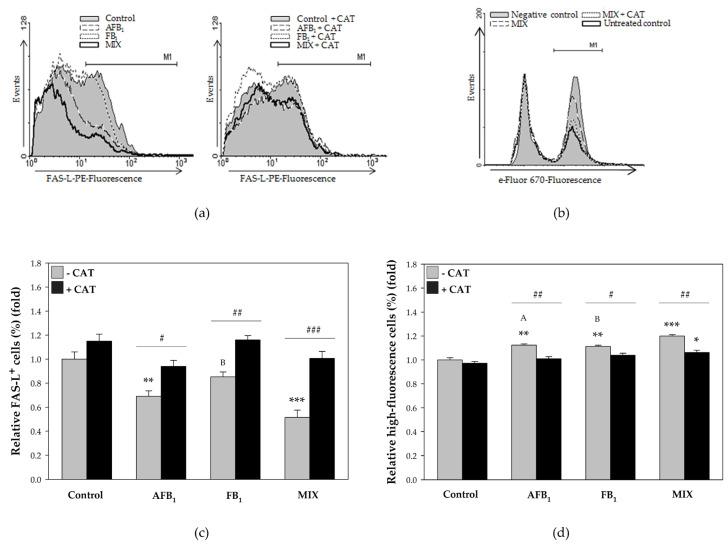
Effects of AFB_1_ and FB_1_ on the cytotoxic capacity of rat SMCs: (**a**) Representative histograms of fluorescence intensity showing surface FAS-L expression on SMCs treated with AFB_1_, FB_1_, or MIX in the absence (left panel) or presence (right panel) of CAT; (**b**) Representative histograms of e-Fluor-670 fluorescence intensity obtained from stained BRL-3A cells (high-fluorescence intensity peak) cultured alone (negative control), co-incubated with untreated SMCs (untreated control), or co-incubated with MIX-treated SMCs in the presence or absence of CAT; (**c**) Percentage of FAS-L^+^ T cells; (**d**) Percentage of BRL-3A cells (high-fluorescence cells). All percentages are expressed relative to the control. In panels (**c**,**d**), gray bars represent cells preincubated with catalase (+ CAT), whereas black bars represent cells without catalase treatment (− CAT); and values are expressed as mean ± SEM. * Indicates differences vs. the corresponding control. Letters indicate differences between the MIX and individual mycotoxin treatments. ^#^ Indicates differences between treatments performed in the presence or absence of CAT. *^,A,#^ *p* < 0.05; **^,B,##^ *p* < 0.01; ***^,###^ *p* < 0.001.

The regulation of the cytotoxic capacity of rat SMCs by mycotoxins and its relationship with their oxidative actions were evaluated by assessing FAS-L surface expression and cytolytic activity against a rat hepatic cell line (BRL-3A) in SMCs cultured in the presence or absence of CAT, AFB_1_, FB_1_, and MIX for up to 48 h. Figure 4a and Figure 4b show representative histograms of the fluorescence intensity of FAS-L and e-Fluor-670, respectively. Figure 4a illustrates FAS-L expression on the surface of SMCs treated with mycotoxins in the absence (left panel) or presence (right panel) of CAT, while Figure 4b shows the BRL-3A cells (high fluorescence intensity peak) that were not lysed by the SMCs. The average percentages of FAS-L^+^ or e-Fluor-670^high^ cells were calculated from these histograms to obtain the results. Flow cytometry analysis indicated that AFB_1_ alone, and particularly in combination with FB_1_, significantly reduced the percentage of FAS-L–expressing T lymphocytes, effects that were prevented by CAT pre-incubation (Figure 4c). Moreover, all mycotoxin treatments significantly increased the percentage of high-fluorescence target cells (BRL-3A) compared with the control, indicating a reduction in the cytolytic activity of SMCs. Among the treatments, MIX exerted the strongest immunotoxic effect. In addition, CAT prevented the mycotoxin-induced impairment of cytolytic activity, as it maintained the proportion of target cells at levels comparable to those observed in control cultures (Figure 4b). Collectively, these results suggest that AFB_1_ and FB_1_, individually and particularly in combination, impair the cytotoxic capacity of SMCs through mechanisms dependent on oxidative stress induction.

### 3.3. Effects of AFB_1_ and FB_1_ on Intracellular Energy Status and Purine Metabolism

**Figure 5:**
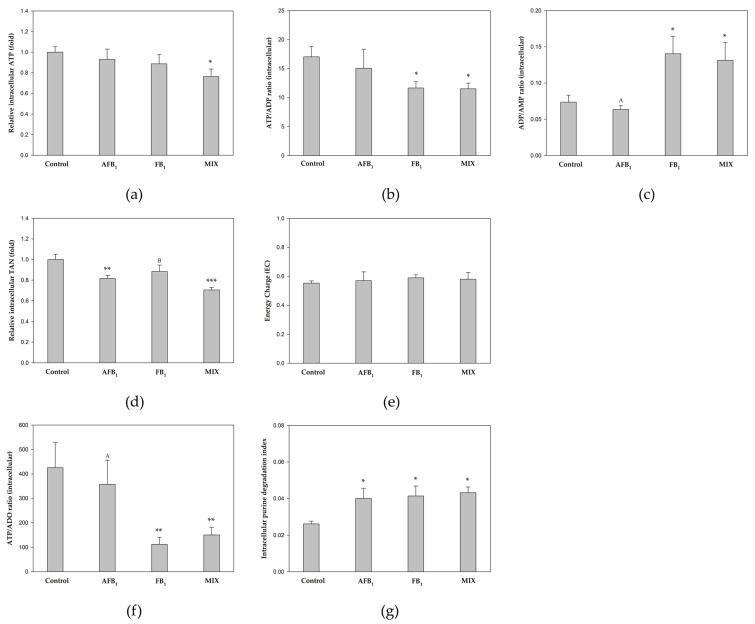
Effects of AFB_1_ and FB_1_ on intracellular energy and purine metabolism in rat SMCs: (**a**) Level of ATP relative to the control; (**b**) ATP/ADP ratio; (**c**) ADP/AMP ratio; (**d**) Level of TAN relative to the control; (**e**) Energy charge (EC); (**f**) ATP/ADO ratio; (**g**) Purine degradation index. Values are expressed as mean ± SEM. * Indicates differences vs. the control. Letters indicate differences between the MIX and individual mycotoxin treatments. *^,A^ *p* < 0.05; **^,B^ *p* < 0.01; *** *p* < 0.001.

To assess the impact of AFB_1_ and FB_1_ on intracellular energy homeostasis in rat SMCs, ATP levels, ATP/ADP and ADP/AMP ratios, total adenine nucleotides (TAN = ATP + ADP + AMP), and energy charge (EC) were determined (Figure 5). The results showed that the MIX significantly reduced ATP levels compared to the control (Figure 5a), and all treatments tended to decrease the ATP/ADP ratio, reaching statistical significance only in the mycotoxin mixture and FB_1_-treated groups (Figure 5b). These treatments also significantly increased the ADP/AMP ratio (Figure 5c). In addition, AFB_1_ and the MIX significantly decreased the size of the intracellular adenine nucleotide pool (TAN) (Figure 5d). However, the EC did not differ significantly among the experimental conditions (Figure 5e). These results suggest that mycotoxin exposure induces a mild to moderate alteration in the intracellular energy balance, characterized by a shift in adenine nucleotide ratios and/or a reduction in the total energy pool, without affecting the overall energy charge. This may indicate the activation of compensatory mechanisms that preserve cellular energy homeostasis.

In parallel, intracellular purine metabolism was examined through the ATP/ADO ratio and the purine degradation index (PDI) in rat SMCs. A significant decrease in the ATP/ADO ratio was observed in the FB_1_ and MIX groups (Figure 5f), whereas all treatments significantly increased the PDI (Figure 5g). These findings suggest an enhanced catabolism of adenine nucleotides under mycotoxin exposure. This response, together with the preserved energy charge, supports the idea of an increased nucleotide turnover, possibly reflecting adaptive mechanisms to maintain cellular energy homeostasis under stress conditions.

### 3.4. Effects of AFB_1_ and FB_1_ on ATP Release and Extracellular Purine Metabolism

**Figure 6:**
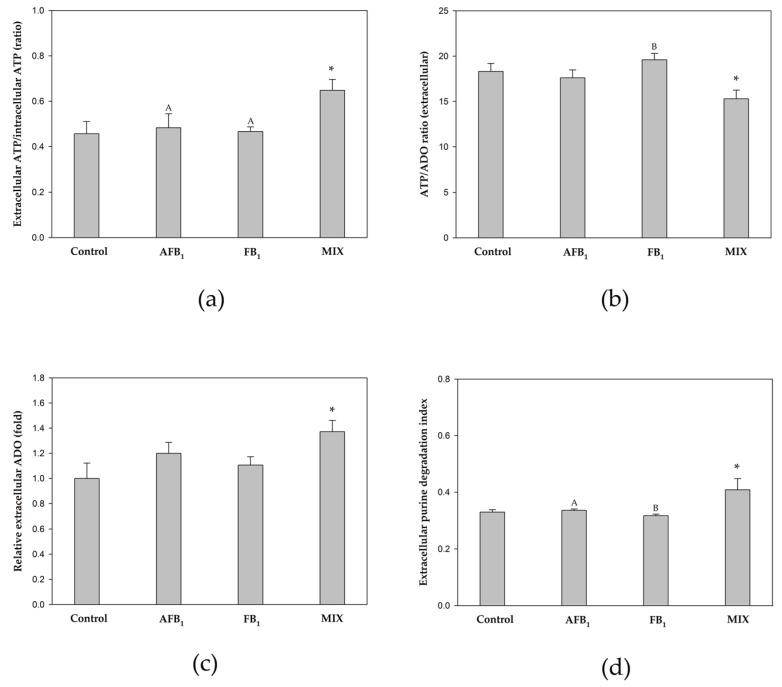
Effects of AFB_1_ and FB_1_ on ATP release and extracellular purine metabolism by rat SMCs: (**a**) ATP release (extracellular ATP/intracellular ATP ratio); (**b**) ATP/ADO ratio; (**c**) Level of ADO relative to the control; (**d**) Purine degradation index (PDI). Values are expressed as mean ± SEM. * Indicates differences vs. the control. Letters indicate differences between the MIX and individual mycotoxin treatments. *^,A^ *p* < 0.05; ^B^ *p* < 0.01.

ATP release into the culture supernatant of rat SMCs was assessed as the extracellular-to-intracellular ATP ratio (Figure 6a). Only the MIX significantly increased this parameter compared to the control and individual mycotoxins. Since neither AFB_1_ nor FB_1_ alone altered ATP release, whereas their combined exposure induced a marked increase, the response suggests that the effect of the mixture exceeded that expected from the individual toxins, indicating an interaction consistent with synergism between AFB_1_ and FB_1_, potentially associated with increased cellular stress or damage induced by the combined exposure.

Extracellular purine metabolism in the culture supernatant of rat SMCs was evaluated by determining the ATP/ADO ratio, relative ADO levels, and the PDI. The MIX significantly decreased the ATP/ADO ratio (Figure 6b) and increased ADO levels (Figure 6c) compared to the control. Furthermore, this treatment was the only condition that significantly increased the PDI (Figure 6d), both relative to the control and to the individual toxins, suggesting an interaction consistent with synergism between AFB_1_ and FB_1_ on extracellular nucleotide catabolism toward adenosine. Together, these findings suggest that combined exposure to both mycotoxins may reflect a coordinated response involving increased ATP release and its subsequent degradation to adenosine, potentially associated with cellular stress and the modulation of extracellular purinergic signaling.

## 4. Discussion

In the present study, it was found that the AFB_1_-FB_1_ mixture exerted marked immunotoxic effects on rat immune cells, characterized by impaired Th1-type responses, reduced cytotoxic capacity, and alterations in energy metabolism and purinergic signaling. Notably, these effects were consistently more pronounced in the combined treatment than in individual exposures. Depending on the endpoint evaluated, the response to the mixture was either consistent with an additive effect, when the combined response approximated the sum of the individual effects, or suggestive of synergism, when the combined response exceeded that expected from the individual mycotoxins. These observations support an interaction between AFB_1_ and FB_1_, as previously suggested for other immunotoxicological parameters [10,23,25,45,47].

One of the central findings is the selective inhibition of Th1-associated cytokines (IFNγ and TNFα) induced by the MIX, while individual mycotoxins showed limited or no effects on their production. This pattern was observed across different immune cell populations, including splenocytes, adherent cells, and intrahepatic lymphocytes, indicating a broad immunosuppressive impact of the mixture. The concomitant increase in IL4–producing NKT cells further suggests a shift from a Th1- toward a Th2-like profile. Such polarization has been associated with impaired antitumor immunity and increased susceptibility to tumor progression [48,49]. In addition, both mycotoxins—especially when combined—impaired the cytotoxic capacity of immune cells, as evidenced by reduced FAS-L expression and decreased lytic activity against target cells. This effect may be mechanistically linked to the suppression of Th1-type cytokines, such as IFNγ and TNFα, which are essential for the activation and/or effector function of cytotoxic lymphocytes [50]. Given the central role of these lymphocytes in tumor surveillance [50,51,52], our findings further support the notion that AFB_1_ and FB_1_ may promote tumor progression not only through genotoxic effects [24] but also by weakening immune-mediated tumor control.

Previous reports have also shown that AFB_1_ can suppress cell-mediated immunity by affecting cytokine production and cytotoxic responses [53], and that FB_1_ exerts immunotoxic effects [4,13], supporting the present findings. However, although several studies have demonstrated that antioxidants mitigate AFB_1_- or FB_1_-induced oxidative stress and cellular damage [9], direct evidence linking ROS modulation to the restoration of specific immune functions, such as Th1 cytokine production and cytotoxic capacity, remains limited. In this context, our findings provide functional support for a causal role of oxidative stress in the immunotoxic effects induced by AFB_1_ and FB_1_. Importantly, the protective effects of catalase on cytokine production and cytotoxic function provide strong evidence for the involvement of oxidative stress in the mechanisms underlying the immunotoxicity induced predominantly by the combined exposure to AFB_1_ and FB_1_, and, to a lesser extent, by individual mycotoxins. This is consistent with previous studies showing that AFB_1_-FB_1_ co-exposure induces a greater disruption of redox homeostasis than either mycotoxin alone in rat SMCs and hepatocytes [24,25], and suggests that oxidative imbalance is a central event in the immunomodulatory action of these toxins [13,25,54].

The ability of catalase to restore IFNγ and TNFα levels and to preserve cytotoxic activity suggests that ROS, particularly hydrogen peroxide, contribute to the immunotoxic mechanisms as signaling mediators, rather than merely reflecting secondary oxidative damage [55]. In this context, ROS are known to modulate nuclear factor-κB (NF-κB)- and MAPK-dependent pathways that play key roles in the regulation of pro-inflammatory cytokines such as IFNγ and TNFα [17], thereby providing a mechanistic basis for the observed suppression of these responses. Consistently, previous reports have shown that ROS can suppress interferon production [56], and decrease the NF-κB-dependent transcription in T lymphocytes [17], and that hydrogen peroxide-mediated signaling inhibits NF-κB activation and TNFα secretion [57]. However, other studies have shown that low microenvironmental levels of ROS can also promote the production of these pro-inflammatory cytokines as well as Th-1 and Th-17 polarizations, reinforcing the notion that ROS act as context- and level-dependent modulators of immune responses [17,58]. Moreover, beyond suppressing Th1-type immunity, redox signaling may also contribute to the promotion of a Th2-polarized phenotype, a process that is further enhanced by excessive microenvironmental ROS [17]. This is consistent with the observed increase in IL4-producing NKT cells, supporting a shift toward a Th2-like profile, which was attenuated by CAT treatment. Altogether, these findings suggest that oxidative stress induced by individual AFB_1_ and FB_1_, and particularly by their combined action, not only impairs cytotoxic and pro-inflammatory responses but also actively reprograms immune function, potentially contributing to a tumor-permissive microenvironment.

Interestingly, the apparent lack of involvement of AhR signaling in the observed inhibition of Th1 differentiation indicates that the immunotoxic effects of AFB_1_ and FB_1_ occurred independently of this pathway. This was despite its known role in regulating both T cell differentiation and CYP1A expression [41], an AFB_1_-biotransforming enzyme and a source of ROS induced by this toxin [25]. The persistence of this effect in cells expressing low-affinity AhR variants further suggests that ROS from AhR-independent pathways likely drove the observed immunosuppression.

Furthermore, this study provides evidence that exposure to individual mycotoxins and especially to their combination, alters the intracellular energy metabolism of splenocytes. The observed decrease in ATP levels and ATP/ADP ratio, together with the increase in the ADP/AMP ratio and the reduction in total adenine nucleotide (TAN) pools, primarily induced by the AFB_1_-FB_1_ mixture, suggests the onset of metabolic stress and increased energy demand. Notably, the preservation of the energy charge (EC) suggests the activation of homeostatic mechanisms that prioritize the maintenance of cellular energetic balance, potentially at the expense of nucleotide pool depletion, as classically described for adenylate energy homeostasis [27]. This is consistent with an increased nucleotide turnover and/or enhanced purine degradation, which promotes the sequential degradation of AMP into metabolites such as inosine, hypoxanthine, and xanthine, with a consequent reduction in the total adenine nucleotide content [20]. In this context, the elevation of the purine degradation index (PDI) and the decrease in the ATP/ADO ratio observed in the present study further support an acceleration of purine catabolism as likely part of an adaptive response to both energetic and oxidative stress. Under conditions of ATP depletion, enhanced AMP degradation contributes to the maintenance of energy homeostasis [59], while purine metabolic pathways are also known to be modulated under oxidative stress conditions, linking nucleotide turnover to redox adaptation [20].

Consistent with the present findings, previous studies have shown that AFB_1_ and FB_1_, when administered individually, may impair mitochondrial function, leading to reduced ATP production and the disruption of cellular bioenergetics, often associated with the induction of oxidative stress [30,31,60]. In contrast, other studies have reported that low concentrations and short exposure times to FB_1_ may prevent mitochondrial membrane potential depolarization and mitigate ATP depletion [61,62]. Moreover, our previous studies in rat SMCs and hepatocytes identified mitochondria as a source of ROS and demonstrated that oxidative stress induces mitochondrial membrane depolarization, both triggered by the AFB_1_-FB_1_ mixture [24,25]. Nevertheless, studies addressing the combined effects of both toxins on energy metabolism remain scarce, and the limited existing co-exposure data are limited by the lack of single-toxin comparisons, hindering the evaluation of interaction effects [63]. Therefore, the present work provides novel insights into the mechanisms underlying the action of the AFB_1_-FB_1_ mixture by integrating intracellular energy metabolism with extracellular purinergic signaling within the same experimental framework. Unlike previous studies that primarily evaluated ATP depletion or mitochondrial dysfunction as isolated indicators of bioenergetic impairment [30,31,60], or specific enzyme activities as indirect indicators of purinergic metabolism [63], the present study directly quantified both intracellular and extracellular adenine nucleotides and their degradation products. This approach enabled the assessment of the overall metabolic outcome of purinergic metabolism and revealed coordinated alterations in intracellular energy status and extracellular purinergic signaling that would not be captured by single-endpoint measurements.

Taking into account the aforementioned evidence, the results of this study suggest that the energetic and metabolic alterations observed in immune cells may be associated with ROS induction. Given that oxidative stress and mitochondrial dysfunction are tightly interconnected processes operating within a self-amplifying loop, they critically shape immune cell metabolism and function. ROS can directly damage mitochondrial lipids, proteins, and DNA, impairing electron transport chain (ETC) activity and reducing ATP production. In addition, dysfunctional mitochondria represent a major intracellular source of ROS due to increased electron leakage, further exacerbating oxidative stress and bioenergetic failure [18,19,21]. This reciprocal relationship is particularly relevant in immune cells, where activation and effector functions are highly energy-dependent. Importantly, disruption of ATP production has been shown to directly inhibit cytokine production and proliferation in T cells [22,64,65]. Therefore, the decrease in intracellular ATP levels and the energy stress induced primarily by the AFB_1_-FB_1_ mixture could compromise T-cell differentiation, activation, and effector function, ultimately impairing immune response.

Furthermore, the combined treatment uniquely increased ATP release into the extracellular space along with enhanced purine degradation, leading to increased ADO accumulation. This is particularly relevant because extracellular ATP acts as a pro-inflammatory danger signal, whereas ADO exerts potent immunosuppressive effects [28], and its accumulation in the tumor microenvironment has been recognized as a key mechanism of immune evasion in HCC [12].

The increase in extracellular ADO levels and the concomitant decrease in the ATP/ADO ratio observed in the culture supernatants of the MIX condition suggest the establishment of an immunosuppressive microenvironment, which may contribute to the reduced Th1 responses and cytotoxic activity. This interpretation is supported by extensive evidence indicating that ADO signaling suppresses T cell activation and effector functions while promoting immune tolerance. In particular, activation of A2A receptors by ADO inhibits Th1 differentiation and pro-inflammatory cytokine production, including IFNγ and TNFα, reduces CD8^+^ T cell cytotoxic programs, and impairs NK cell activity, while enhancing regulatory pathways such as Treg induction and anti-inflammatory cytokine production, thereby favoring an immunosuppressive environment [28,66,67,68,69]. Moreover, ADO has been reported to promote Th2 differentiation and cytokine production (IL4, IL10, IL13) [70], reprogram macrophages from a pro-inflammatory M1 phenotype characterized by TNF-α and IL6 production toward an anti-inflammatory M2 phenotype, and modulate antigen-presenting cells by decreasing IL12 and increasing IL10 or IL4 production, thereby limiting Th1 polarization and favoring a shift toward Th2 responses [28].

Taken together, these findings support a model in which combined exposure to AFB_1_ and FB_1_ induces oxidative stress in immune cells, leading to alterations in intracellular energy metabolism and extracellular purinergic signaling. These interconnected mechanisms contribute to the suppression of Th1 responses and cytotoxic immune functions, while promoting a Th2-biased immune profile and facilitating immune evasion. To the best of our knowledge, this is the first study to integrate these mechanisms within a single experimental framework, providing novel insight into how AFB_1_-FB_1_ co-exposure may impair antitumor immunity and promote tumor progression beyond their well-established genotoxic effects. The interactions observed between AFB_1_ and FB_1_, ranging from additive to synergism-consistent responses depending on the endpoint evaluated, further highlight the importance of considering co-exposure scenarios in risk assessment, as mycotoxins are frequently found in combination in natural settings [1,2,9].

Future studies should further elucidate the molecular pathways linking oxidative stress to purinergic signaling and immune dysfunction, as well as the potential involvement of ectonucleotidases and adenosine receptors in mediating these effects. In addition, it will be important to determine whether these mechanisms also contribute to immunotoxic responses at lower mycotoxin concentrations.

## 5. Conclusions

The present study demonstrates that combined in vitro exposure to AFB_1_ and FB_1_ exerts pronounced immunotoxic effects on rat immune cells, characterized by the suppression of Th1-type responses, impairment of cytotoxic function, and a shift toward a Th2-like immune profile. These effects were consistently more marked under co-exposure conditions, supporting interactions between both mycotoxins that were compatible with additive effects for some endpoints and suggestive of synergism for others.

Mechanistically, the findings indicate that oxidative stress plays a central role in mediating these alterations, as evidenced by the protective effects of catalase on cytokine production and cytotoxic activity. In addition, the disruption of intracellular energy metabolism and the shift in purinergic signaling, marked by increased extracellular adenosine accumulation, emerge as key interconnected processes contributing to immune dysfunction.

Altogether, these results provide new insights into the mechanisms underlying the immunotoxicity of AFB_1_-FB_1_ co-exposure and highlight the importance of considering combined mycotoxin exposure in risk assessment, given its potential to promote immunosuppression and compromise antitumor immune responses.

## Data Availability

The data that support the findings of this study are available from the corresponding authors upon reasonable request.

## Supplementary Materials

The following supporting information can be downloaded at: https://www.mdpi.com/article/10.3390/jof12070520/s1, Figure S1: Effects of AFB_1_ and FB_1_ on TNFα Secretion by rat ACs: Level of TNFα in AC culture supernatants. Values are expressed as mean ± SEM. * Indicates differences vs. the control. Letters indicate differences between MIX and individual mycotoxin treatments. **,^B^ *p* < 0.01; ***,^C^ *p* < 0.001.

## Abbreviations

The following abbreviations are used in this manuscript:

ADO	Adenosine
ADP	Adenosine diphosphate
AFB_1_	Aflatoxin B_1_
AFBO	AFB_1_-exo-8,9-epoxide
AMP	Adenosine monophosphate
ATP	Adenosine triphosphate
CAT	Catalase
CYP	Cytochrome P450
FAS-L	FAS ligand
FB_1_	Fumonisin B_1_
IFNγ	Interferon gamma
IHLs	Intrahepatic leukocytes
MIX	AFB_1_-FB_1_ mixture
NK	Natural killer cells
NKT	Natural killer T lymphocytes
ROS	Reactive oxygen species;
SMCs	Spleen mononuclear cells
TNFα	Tumor necrosis factor alpha
