# Supplementary figures and images for: Purinergic and Energy Metabolism Disruption in Oxidative Stress-Mediated Immunotoxicity Induced by Aflatoxin B1 and Fumonisin B1
Source: J Fungi (Basel). 2026 Jul 15;12(7):520. doi: 10.3390/jof12070520 (PMC13412577; doi:10.3390/jof12070520)

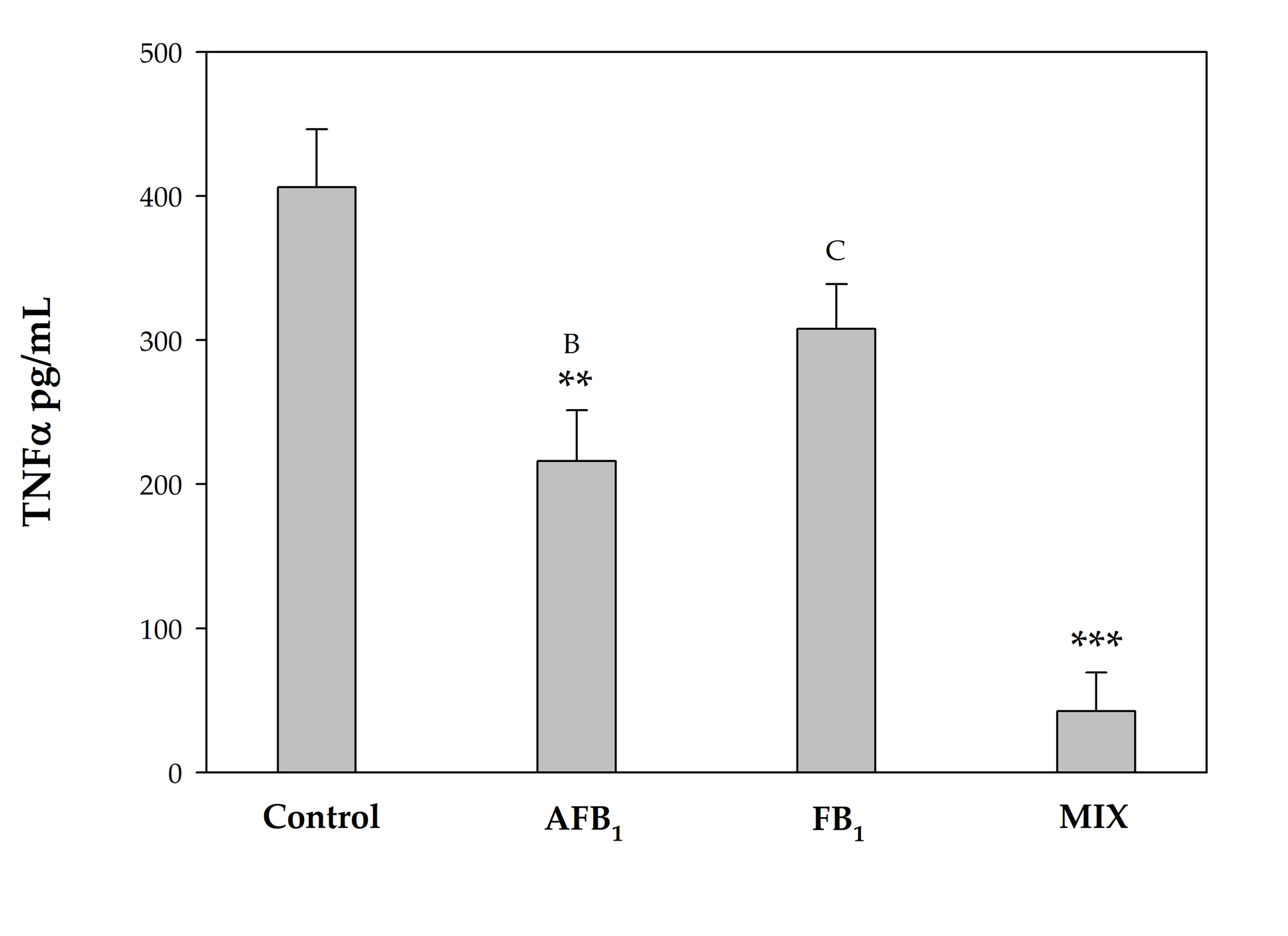

Supplement: Supplementary file 1 [file jof-12-00520-s001.zip › Figure S1. Effects of AFB1 and FB1 on TNFα Secretion by rat ACs.tif]
